# Evaluation of sepsis CDS tool knowledge and utilization among graduate medical trainees: insights to inform redesign in an academic health system

**DOI:** 10.3389/fmed.2025.1660390

**Published:** 2026-01-05

**Authors:** Shadi Hijjawi, Paddy Ssentongo

**Affiliations:** 1 Department of Medicine, Penn State Health Milton S. Hershey Medical Center, Hershey, PA, United States; 2 Division of Infectious Diseases and Epidemiology, Department of Medicine, Penn State Health Milton S. Hershey Medical Center, Hershey, PA, United States; 3 Department of Public Health Sciences, The Pennsylvania State University College of Medicine, Hershey, PA, United States

**Keywords:** clinical decision support, sepsis, alert fatigue, medical education, workflow integration

## Abstract

**Background:**

Early recognition and treatment of sepsis is critical to reducing mortality. Many hospitals have adopted Electronic Health Record (EHR)-based clinical decision support (CDS) tools to assist with timely identification and management of sepsis. However, over-alerting and poor integration into clinician workflow may limit their effectiveness.

**Objective:**

To assess medical trainees’ awareness, usage, and perceptions of two EHR-integrated sepsis CDS tools—a sepsis alert and sepsis advisor—at a tertiary academic hospital to inform future system-wide sepsis workflows redesign.

**Methods:**

A cross-sectional survey was distributed to 673 residents and fellows at Penn State Milton S. Hershey Medical Center in March 2022. The anonymous survey explored familiarity with sepsis tools, usage patterns, perceived usefulness, and suggestions for improvement.

**Results:**

Ninety-two trainees responded (13.7% response rate). Among 80 who recognized the sepsis alert, only 11.3% had used the advisor. Alert fatigue (62.5%) and limited clinical utility (46.3%) were major concerns. Only 16.3% felt both tools were helpful, while 52.5% found them unhelpful. Lack of awareness about advisor features and alert suppression mechanisms further limited engagement. Respondents who found the tools unhelpful recommended a non-interruptive, asynchronous alert format, a “patient already being treated” option, and redesigning the advisor into a streamlined, workflow-aligned tool.

**Conclusion:**

While sepsis CDS tools offer potential for improving care, their impact is limited by knowledge gaps, usability issues, alert fatigue, and poor clinical fit. Addressing these issues through better design, education, and workflow integration—guided by frontline user input—should be a core strategy in CDS quality improvement efforts.

## Highlights

- Many medical trainees find existing sepsis alerts disruptive and unhelpful. Redesigning tools to align with clinical workflow and reducing alert fatigue may improve usability and impact.

## Introduction

Sepsis is a life-threatening condition and a leading cause of mortality among hospitalized patients in the United States (1). It is estimated that about 1.7 million Americans develop sepsis each year, and up to 270,000 die as a result (1). Prompt recognition and treatment of sepsis are critical for survival as mortality increases by approximately 8% with each hour of delayed treatment initiation (2). The 2021 Surviving Sepsis Campaign guidelines strongly recommend routine sepsis screening as a best-practice strategy to improve early recognition and timely treatment (3). To improve early detection and management, many hospitals have implemented electronic sepsis alerts and clinical decision support (CDS) tools within the electronic health record (EHR) (4). These CDS alerts aim to notify providers of potential sepsis in real time and prompt timely action. They also reinforce adherence to core sepsis treatment elements, including monitoring lactate trends, obtaining blood cultures, rapid antibiotics administration, adequate intravenous fluid resuscitation, and maintaining hemodynamic stability. However, a major challenge with sepsis alert systems is their tendency to favor sensitivity over specificity, resulting in numerous false-positive alerts (5, 6). In practice, this means that alerts flag many patients who ultimately do not have sepsis or who have already been clinically recognized, which can lead to alert fatigue among clinicians (7). When providers become inundated with frequent alerts, they may start to ignore or override them, undermining the alert’s purpose. In addition, the usability and workflow integration of these tools are critical factors for adoption: if an alert or advisory prompt is cumbersome, disruptive, or not perceived as helpful, busy clinicians (especially trainees managing many tasks) may be reluctant to use it. Thus, understanding frontline clinicians’ experiences with and attitudes toward sepsis alerts is essential to identify why such decision support tools might fail and how they can be improved. In addition, sepsis care has become a national pay-for-performance priority through the Centers for Medicare & Medicaid Services SEP-1 (Severe Sepsis and Septic Shock Management Bundle) measure, further emphasizing the importance of reliable and usable sepsis CDS tools (8).

Our academic hospital implemented two sepsis-related CDS tools. The first is an EHR-integrated pop-up alert for suspected sepsis, and the other is a sepsis advisory tool that provides evidence-based interventions to treat the sepsis. This survey represents the initial diagnostic phase of a larger institutional performance-improvement project guided by the SQUIRE framework aimed at redesigning sepsis CDS tools and workflows.

### Objective

We undertook this study to formally evaluate trainees’ knowledge, usage, and perceptions of sepsis tools as a preliminary step in a system-wide initiative to review and enhance sepsis workflows and decision support systems.

## Materials and methods

### Study design and participants

We conducted a cross-sectional survey of medical trainees at Penn State Milton S. Hershey Medical Center, a tertiary academic hospital serving 27 counties with approximately 1.6 million people in Central and Eastern Pennsylvania, in March 2022. The target population was graduate medical trainees, resident and fellow physicians, across specialties at the institution (*N* = 673 trainees in 2022). An email explaining the study and containing a link to an anonymous online questionnaire was sent to all trainees. Participation was voluntary, and no incentives were provided. Completion of the survey implied consent to participate. The survey was designed as a quality improvement initiative to inform internal practice; responses were collected without any personal identifiers. The institutional review board was consulted and determined that this project qualified as exempt quality improvement, not human subjects research.

### EHR tools

Penn State Hershey Medical Center is using Cerner Millennium (Oracle Health) EHR (Cerner Corporation, Kansas City, MO, acquired in 2022 by Oracle Health, Austin Texas). The EHR is equipped with a cloud-based CDS tool that continuously screens patients for meeting Systemic Inflammatory Response Syndrome (SIRS) criteria, severe sepsis or septic shock.

**FIGURE 1:**
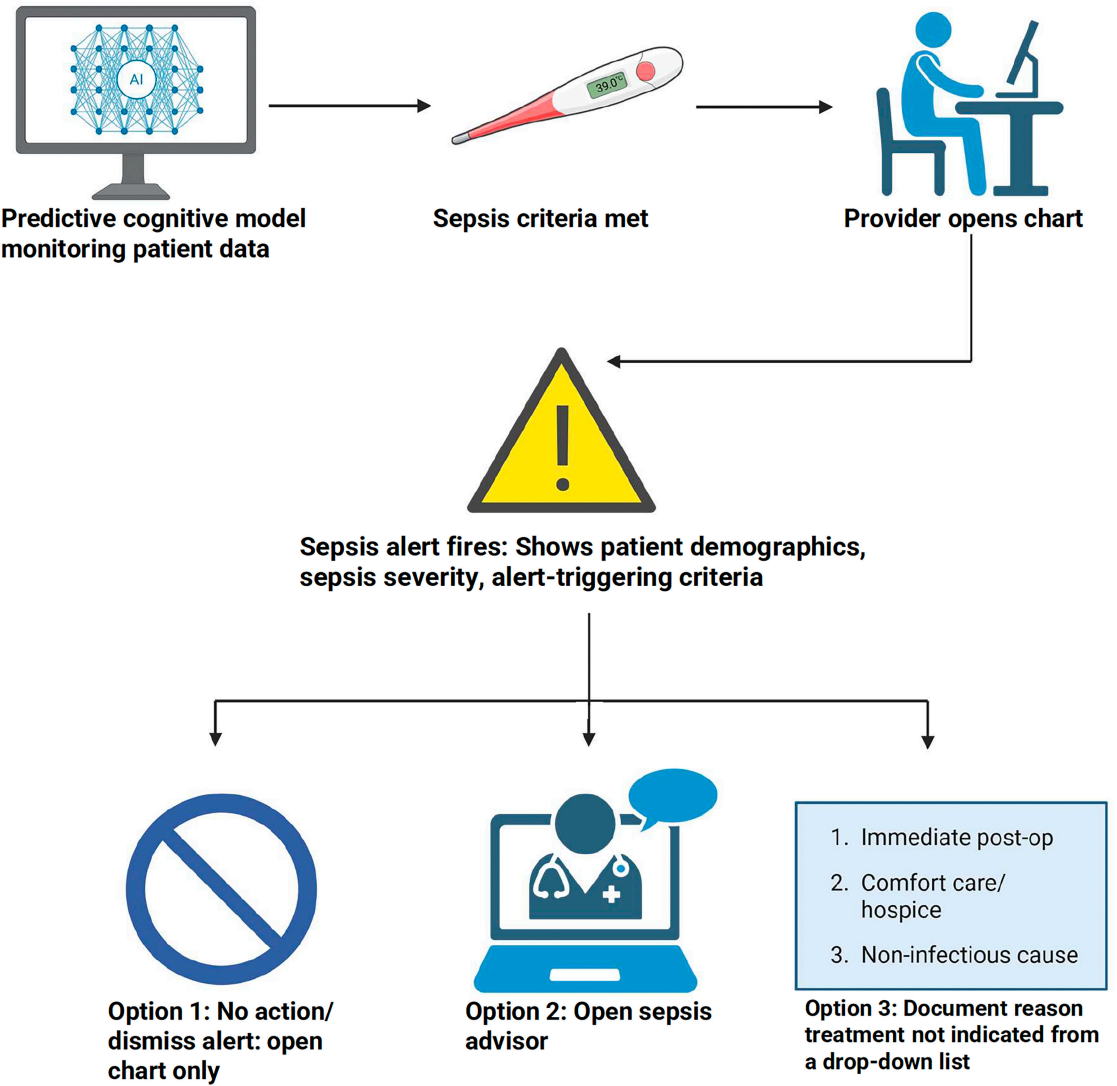
Flow diagram of the sepsis alert workflow showing data monitoring, alert firing, and three provider response options within the EHR. Created with BioRender.com.

The sepsis alert functioned identically in both the Emergency Department and inpatient settings and applied only to adult patients. The sepsis alert was set up to open-chart alert that pops every time a provider opens an adult patient’s chart, and the criteria are met (Figure 1). The alert was designed based on the St. Johns sepsis agent (9), which presents the sepsis criteria that trigger the alert and gives a few options to proceed. Options are either opening the chart without any action, opening the sepsis advisor, or documenting why the patient does not meet sepsis criteria or does not need treatment by selecting one of a few given options for that. Documentation options include suspect non-infectious cause, patient is immediately post op or intend to write comfort care/hospice orders. None of these options indicates whether selecting it will suppress the alert even though selecting to document a reason for treatment not indicated or selecting and signing the orders in the advisor would have suppressed the alert for a certain period.

The sepsis advisor is a comprehensive CDS tool that provides relevant information about the patient in terms of recent blood tests and positive cultures, listed allergies, currently ordered antibiotics, and system-based options of antimicrobial regimens that are supported by the antimicrobial stewardship for treatment. In addition, it has options for ordering labs, microbiology, intravenous (IV) fluids and vasopressors. It also provides an option to add sepsis diagnosis to the problem list.

Providers are expected to update problem list and provide appropriate documentation in their notes in the EHR using a standard system-wide auto-text. This auto-text is a feature in the Cerner Millennium EHR that allows clinicians to insert frequently used phrases, templates or entire documentation blocks using customized shortcuts to enhance documentation efficiency and consistency across different users.

### Survey instrument

The survey was developed by the study team and included both multiple-choice questions and in some questions open-ended response fields. Some questions offered only one selection, whereas others offered multiple selections. Besides basic demographic questions, key content domains covered in the questionnaire were:

- *Awareness and knowledge of the sepsis tools*: Trainees were asked if they were aware of the sepsis alert and the sepsis advisor tools in the EHR. Snapshot pictures of the tools were provided in the related questions to simplify the identification of the tools in question and confirm familiarity. We also asked if they knew specific functions of the tools (for example, that the sepsis advisor could be manually launched via the order entry menu).
- *Usage, overall experience, perceptions of usefulness and usability*: Questions assessed how often the trainees use the sepsis advisor when managing patients and whether trainees found the sepsis alert and advisor helpful to their clinical workflow or not. We offered structured multichoice selections to provide feedback on user-friendliness (frequency of alerts, contribution to alert fatigue, ease of use, clinical relevance, and appropriate workflow, etc.) and perceived impact on patient care. Participants who indicated that the tools were unhelpful were prompted to select reasons why (e.g., “wrong workflow,” “cumbersome to use,” “alert is redundant,” etc.).
- *Suggestions for improvement:* Respondents with “not useful” perception of the tools were asked to select from a list of potential suggestions for improvement that might make them more likely to use it. Multichoice selections in addition to free-text input were offered. Likert-scale items were included to gauge degree of agreement in a planned intervention to improve sepsis CDS tools.
- *Sepsis care education and documentation*: Trainee participants were asked if they received any training or education in sepsis management and sepsis care documentation.

Additionally, we asked about any general suggestions to improve sepsis care and education for trainees.

The final anonymized survey was administered via a secure online survey platform. Two reminder emails were sent at 1-week intervals to boost response rates.

### Data collection and analysis

Survey responses were collected electronically and exported for analysis. For quantitative multiple-choice items, we calculated the frequencies and percentages of respondents selecting each response option. Descriptive statistics (counts and percentages) are presented for the main outcome measures. In calculating percentages, in the most part the denominator was typically the total number of survey respondents (*N* = 92). But for certain items that were conditional, for example only those who were aware of the sepsis alert who were asked certain follow-up questions based on their knowledge of the sepsis tools, we used the appropriate subgroup count as the denominator and specify this where relevant. Specifically, questions regarding the knowledge and the use experience of the sepsis tools were extended only to those who reported being familiar with the sepsis alert (80 respondents). In addition, those who reported that the sepsis alert and advisor are helpful as they are (*n* = 13) were excluded from the questions about why the tools are unhelpful and suggestions for improvement; so percentages for those items reflect the proportion of the 67 respondents who provided an opinion on that. For questions that allowed multiple selections, for example suggestions on how to improve the sepsis tools, all chosen options were tallied.

All analyses were conducted as part of a quality improvement review; no formal statistical hypothesis testing (comparative statistics) was performed, given the descriptive and exploratory nature of the survey. Analyses were conducted in R statistical language version 4.2.3 (R Foundation for Statistical Computing, Vienna, Austria) (10).

## Results

### Sepsis tools survey respondent characteristics

Of the 673 trainees invited, 92 responded, yielding a response rate of 13.7%. This included residents and fellows from a variety of departments. We did not collect detailed demographics in order to keep the survey brief and anonymous. Demographic details are presented in Table 1. Most participants were affiliated with the departments of Internal Medicine and Pediatrics. Because the sepsis alert fires only for adult patients, pediatric trainees—who comprised the second-largest respondent group (17/92)—were less likely to encounter the alert in routine practice. Consistent with this, four of the pediatric respondents reported unfamiliarity with the alert, and the remaining pediatric trainees who were familiar with it indicated that they never used the advisor.

**TABLE 1:** Demographic characteristics of survey respondents (*N* = 92).

Characteristic	Category	n (%)
Sex	Male	47 (51.1)
	Female	42 (45.6)
	Prefer not to say	3 (3.3)
Age (years)	20–29	38 (41.3)
	30–39	50 (54.3)
	40–49	4 (4.3)
Postgraduate year (PGY) training level	PGY1	24 (26.1)
	PGY2	20 (21.7)
	PGY3	17 (18.5)
	PGY4	17 (18.5)
	PGY5	6 (6.5)
	PGY6	6 (6.5)
	PGY7	1 (1.1)
	PGY8	1 (1.1)
Department	Internal medicine	25 (27.2)
	Pediatrics	17 (18.5)
	Anesthesia	9 (9.8)
	Family medicine	6 (6.5)
	Psychiatry	5 (5.4)
	Medicine-pediatrics	4 (4.3)
	Emergency medicine	4 (4.3)
	Radiology	4 (4.3)
	General surgery	3 (3.3)
	Urology	3 (3.3)
	Otolaryngology	3 (3.3)
	Others	9 (9.8)
Trainee type	Resident	72 (78.3)
	Fellow	20 (21.7)

Sex distribution was close to even, with 51.1% identifying as male. Most respondents (54.3%) were between the ages of 30 and 39 years. Training levels ranged from PGY (Postgraduate Year) 1 to PGY8, with PGY1–PGY4 comprising the majority. Overall, 78.3% of respondents were residents and the rest were fellows.

### Awareness, usage and experience of sepsis CDS tools

Most respondents, 80/92 (87%) were aware of the sepsis alert notification in the EHR. The remaining 12 respondents were either not aware, not sure or did not work inpatient. Out of the 80 respondents who were aware of the alert, 9 (11.3%) indicated that they had at some point used the sepsis advisor but rarely when they get the alert. The remainder did not report using the advisor, including 23/80 (28.8%) who indicated they were not aware that they could launch it from the alert. On the other hand, few respondents were aware of the advisor’s features since 17/80 (21.3%) knew that it could be proactively launched from the order entry menu.

Table 2 is summarizing survey responses from trainees familiar with the sepsis alert, detailing their awareness, usage patterns, perceptions of usefulness, and reasons cited for finding the tools unhelpful.

**TABLE 2:** Awareness, use, experience, and perceived utility of sepsis clinical decision support tools among trainees (*N* = 80, unless otherwise noted).

Domain	Question	Responses n/N (%)
Awareness, use, and overall experience	Aware of sepsis alert	80/92 (87)
	Unaware of options to suppress sepsis alert	36/80 (45)
	Unaware of sepsis advisor existence	17/80 (21.3)
	Unaware of ability to launch sepsis advisor from sepsis alert	23/80 (28.8)
	Aware of ability to launch sepsis advisor from order entry menu	17/80 (21.3)
	Used sepsis advisor	9/80 (11.3)
	Reported current tools are helpful and easy to use	4/80 (5)
	Reported excessive alerting causing alert fatigue	50/80 (62.5)
	Received additional alerts by nursing staff that patient had sepsis alert	27/80 (33.8)
	Perceived overall limited clinical utility	37/80 (46.3)
Perceptions of usefulness	Both tools helpful	13/80 (16.3)
	Both tools unhelpful	42/80 (52.5)
	Advisor helpful, alert unhelpful	20/80 (25)
	Alert helpful, advisor unhelpful	5/80 (6.3)
	Sepsis advisor helpful	33/80 (41.3)
	Sepsis advisor unhelpful	47/80 (58.7)
	Sepsis alert helpful	18/80 (22.5)
	Sepsis alert unhelpful	62/80 (77.5)
Reasons for unhelpfulness (*N* = 67)	Wrong workflow (open-chart alert)	34/67 (50.7)
	Redundant alerts (patient already on sepsis treatment)	46/67 (68.7)
	Wrong clinical context (alert on non-septic patient)	50/67 (74.6)
	Frequent sepsis alerts	45/67 (67.2)
	Sepsis advisor is cumbersome to use	20/67 (29.9)
	Sepsis advisor is missing important orders/labs	4/67 (6)

### Perceptions of sepsis tools usefulness

Trainees’ opinions on the sepsis alert system’s usefulness were mixed but tended toward negative. Table 2 summarizes the distribution of perceptions regarding helpfulness of these tools. In brief, 13/80 (16.3%) of respondents felt that both the sepsis alert and the sepsis advisor were helpful as they currently exist. A much larger proportion—42/80 (52.5%) of respondents—felt that sepsis alert and advisor tools were unhelpful. The remaining respondents had a nuanced view; about 20/80 (25%) said that the sepsis advisor was helpful, but the alert itself was not, conversely 5/80 (6.3%) found the alert helpful but thought the advisor was not helpful. The total number of respondents who said the sepsis advisor was helpful was 33/80 (41.3%) while for the alert was 18/80 (22.5%).

### User-reported barriers to effective alert and advisor use

Among the 67 trainees who found the sepsis alert and/or advisor unhelpful, the most frequently cited reason was poor clinical context—74.6% reported that the tools often triggered alerts for patients who were clearly not septic. A majority (68.7%) also indicated that the alert was redundant because septic patients already had appropriate orders in place, while 67.2% experienced excessive alerting. Half of respondents (50.7%) felt that triggering the alert upon chart opening disrupted clinical workflow. Additional concerns included the tool being too cumbersome to use efficiently (29.9%) and lacking relevant orders or labs (6%), highlighting broad usability and timing issues.

### Suggestions for sepsis tools improvement

**FIGURE 2:**
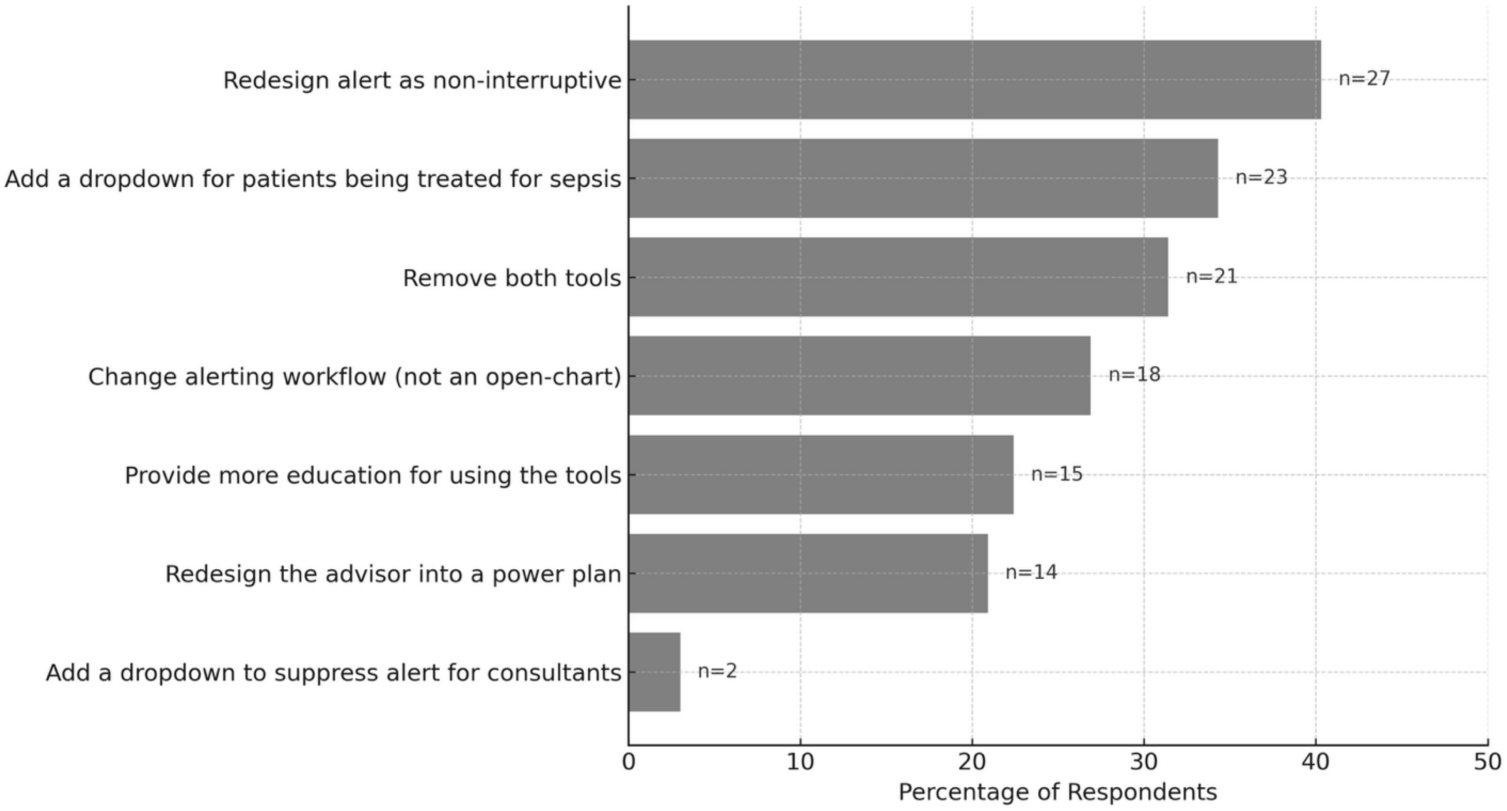
Suggestions for improving sepsis alert and advisor tools (*N* = 67). Horizontal bar plot showing the percentage of respondents suggesting various improvement suggestions. Raw counts (n) are displayed next to each bar.

The same group of respondents who said the tools were unhelpful were asked to give suggestions for improvement (Figure 2). The most common recommendation was to convert the alert into a non-interruptive, asynchronous format (40.3%), followed by adding a dropdown option indicating the patient is already being treated for sepsis (34.3%), and completely removing both the alert and advisor (31.4%). Other suggestions included modifying when the alert is triggered, enhancing user education, and redesigning the advisor into a streamlined PowerPlan (order set). When asked if they agree with the temporary plan to inactivate the sepsis alert, 88% (59/67) agreed or strongly agreed with that and the rest except 1 were neutral to that plan (Table 3). Finally, 54.4% (50/92) agreed that a well-designed and implemented sepsis CDS tool could enhance patient care.

**TABLE 3:** Agreement with temporary plan to inactivate sepsis alert (*N* = 67).

Agreement with temporary plan to inactivate alert	n (%)
Agreed or strongly agreed	59 (88)
Neutral	7 (10.5)
Disagreed	1 (1.5)

Responses are shown as raw counts with percentages in parentheses.

### Sepsis education and documentation

**FIGURE 3:**
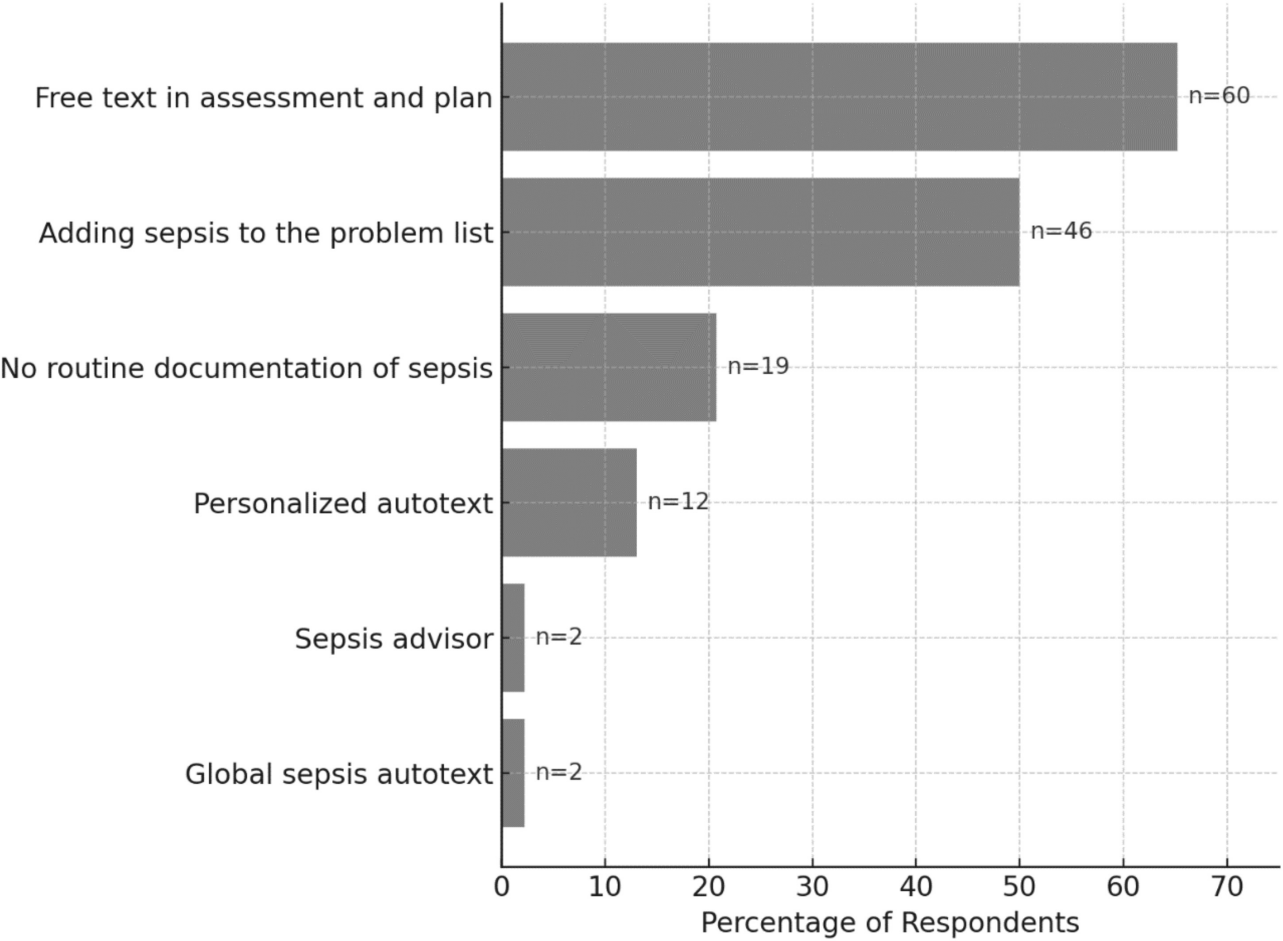
Sepsis documentation practices (*N* = 92). Horizontal bar plot showing the percentage of respondents for various documentation practices. Raw counts (n) are displayed next to each bar.

Lastly, when asked about receiving education on sepsis management, sepsis bundle, and documentation, 54.4% (50 out of 92) reported receiving this education during onboarding or training. Regarding familiarity with documenting sepsis care, including sepsis bundle requirements, approximately two-thirds [65.2% (60/92)] felt confident doing so, with around half of them (32 out of 60) indicated they could benefit from a refresher. The majority preferred free texting their assessment and plan [65.2% (60/92)] or adding sepsis to the problem list [50.0% (46/92)]. Use of personalized autotext [13.0% (12/92)], the global sepsis autotext [2.2% (2/92)], and the sepsis advisor [2.2% (2/92)] were limited. Notably, 20.7% (19/92) of respondents reported not routinely documenting sepsis at all. Sepsis documentation practices are summarized in Figure 3.

## Discussion

Our findings offer a revealing snapshot of how trainees experience and perceive CDS tools for sepsis in a real-world academic environment. With a response rate of 13.7%, our sample reflected a reasonable cross-section of residency and fellowship programs, dominated by Internal Medicine and Pediatrics. The data show that while awareness of the sepsis alert is high (87%), meaningful engagement with its companion integrated tool—the sepsis advisor—is strikingly low.

This disparity between awareness and usage speaks volumes. Just under half (45%) of those aware of the sepsis alert did not know the options to suppress it, nearly one-third of them did not know they could launch the sepsis advisor from within the alert, one-fifth did not know the advisor exists, and only 11.3% had used it at all—most of them infrequently. This aligns with prior studies that show poor integration of CDS tools into clinical workflow significantly diminishes adoption (11). Tools that are perceived as intrusive, redundant, or misaligned with the timing of clinical decision-making are often underutilized or dismissed altogether (12).

Alert fatigue emerged as the most common complaint, cited by 62.5% of respondents. This is consistent with extensive literature showing that excessive or poorly timed alerts erode attention and desensitize users to potentially important warnings (13, 14). In our sample, this fatigue was compounded by perceptions of low clinical utility (46.3%), poor contextual relevance (74.6%), and lack of workflow alignment (50.7%).

Perhaps more telling is the deep ambivalence about whether these tools help at all. Only 16.3% felt both the sepsis alert and advisor were helpful in their current form. Over half (52.5%) felt that neither was helpful. This skepticism is not unique to our institution; other studies have shown similar patterns when sepsis CDS systems lack personalization, flexibility, or integration with clinician mental models (15, 16).

Usability also emerged as a major determinant of tool disengagement. Many respondents found the tool unhelpful due to being prompted during the wrong workflow at the time of opening the chart. In addition, the sepsis advisor requires multiple steps to initiate care, which many trainees found burdensome amid busy clinical workflows. The sepsis advisor’s complex interface discouraged engagement, with trainees preferring instead a more streamlined, one-click solution for initiating sepsis protocols—an approach echoed in recent literature highlighting the importance of user-friendly CDS tools in sepsis identification and response (17).

When asked for improvements, the majority of respondents did not advocate for elimination, but for redesign. A non-interruptive, asynchronous alert format was the most popular solution (40.3%), echoing the literature on passive CDS tools being less disruptive and more acceptable to users. Similarly, suggestions like adding a “patient already being treated for sepsis” dropdown or converting the advisor into a streamlined PowerPlan (order set) reflect a desire for tools that align with clinical realities, not idealized workflows. In addition, these suggestions reflect known strategies for CDS success (6). A non-interruptive, asynchronous alert refers to a passive signal, such as a color-coded banner or icon, visible within clinician workflow that does not disrupt chart navigation. This model avoids pop-ups, allows clinicians to engage the alert voluntarily, and is increasingly favored to minimize alert fatigue.

Despite these criticisms, more than half of the respondents agreed that a well-designed sepsis CDS tool could meaningfully improve care. Also, only 54% indicated that they received education about sepsis during onboarding, and of those that felt confident documenting sepsis care, more than half indicated they could benefit from a refresher. This suggests that the problem is not necessarily with the tool itself, but with the implementation of it. The opportunity here is to build on what we’ve learned, particularly the importance of human-centered design, user education, and intelligent alert suppression mechanisms (6, 18).

Finally, our findings on sepsis documentation underscore the challenge of practice variation. While most respondents report confidence in documenting sepsis, few utilize standardized tools such as autotext or the sepsis advisor. Instead, documentation tends to rely on free-text entries (65%) and problem list updates (50%)—the latter being somewhat more structured but still lacking the consistency of formal documentation tools. This highlights a tension between the need for structured data capture and the desire for clinical expressiveness, one that any future redesign must carefully address.

In summary, trainees are not disengaged; they are discerning. Their feedback provides a clear roadmap for CDS governance to reduce noise, improve relevance, support workflow, and earn trust. This physician-based data informed and guided the next iteration of our system-wide efforts to improve sepsis workflows and CDS tools as part of an institutional governance strategy in the EHR.

### Limitations and strengths

Several limitations should be noted. As with most single-center descriptive surveys, risks of response bias and limited generalizability are substantial. The modest response rate (13.7%) introduces the potential for selection bias, as trainees with strong opinions may have been more likely to respond. As a single-center study using a specific EHR platform (Cerner Millennium), findings may not generalize to other institutions or systems. Additionally, reliance on self-reported data introduces the risk of recall bias. Objective EHR-based usage metrics and multi-site validation would enhance future studies. In addition, we did not analyze potential differences by specialty, training year, or prior sepsis training, which might influence responder attitudes. The survey occurred in March 2022, near the end of the COVID-19 pandemic, when sepsis-like presentations were common; this context may have influenced trainee perceptions of sepsis alerts and documentation.

Nonetheless, the study has important strengths. It directly captures the voice of frontline trainees—an underrepresented group in CDS evaluations—highlighting user-centered barriers and solutions. These insights are especially valuable for health systems seeking to advance CDS adoption through QI initiatives. This feedback has informed our upcoming changes in the EHR-based sepsis CDS tools. By combining quantitative and qualitative data, the study offers a nuanced understanding of how design, education, and workflow integration interact to influence CDS effectiveness.

## Conclusion

Sepsis CDS tools hold promise for improving early detection, intervention and management of a life-threatening condition, but their effectiveness is contingent upon clinician engagement. This study identifies knowledge deficits, usability challenges, and limited clinical relevance as major barriers to adoption. Addressing these through improved user-centered design, targeted education, and workflow-aligned integration is essential. Our operational recommendations complement the Surviving Sepsis Campaign’s 2023 Research Priorities, which emphasize improving sepsis recognition and predictive modeling, but do not address practical CDS implementation challenges such as workflow integration and human-centered design. Engaging frontline users in design, effectiveness and usability evaluation of these tools should be central to CDS governance strategy in quality improvement efforts.

## Data Availability

The original contributions presented in this study are included in this article/Supplementary material, further inquiries can be directed to the corresponding author.
