# Correction: Evaluation of sepsis CDS tool knowledge and utilization among graduate medical trainees: insights to inform redesign in an academic health system

**DOI:** 10.3389/fmed.2026.1788147

**Published:** 2026-03-02

**Authors:** Shadi Hijjawi, Paddy Ssentongo

**Affiliations:** 1 Department of Medicine, Penn State Health Milton S. Hershey Medical Center, Hershey, PA, United States; 2 Division of Infectious Diseases and Epidemiology, Department of Medicine, Penn State Health Milton S. Hershey Medical Center, Hershey, PA, United States; 3 Department of Public Health Sciences, The Pennsylvania State University College of Medicine, Hershey, PA, United States

**Keywords:** clinical decision support, sepsis, alert fatigue, medical education, workflow integration

There was a mistake in Figure 1 as published. The wrong version of the figure was used. The corrected Figure 1 appears below.

**Figure 1:**
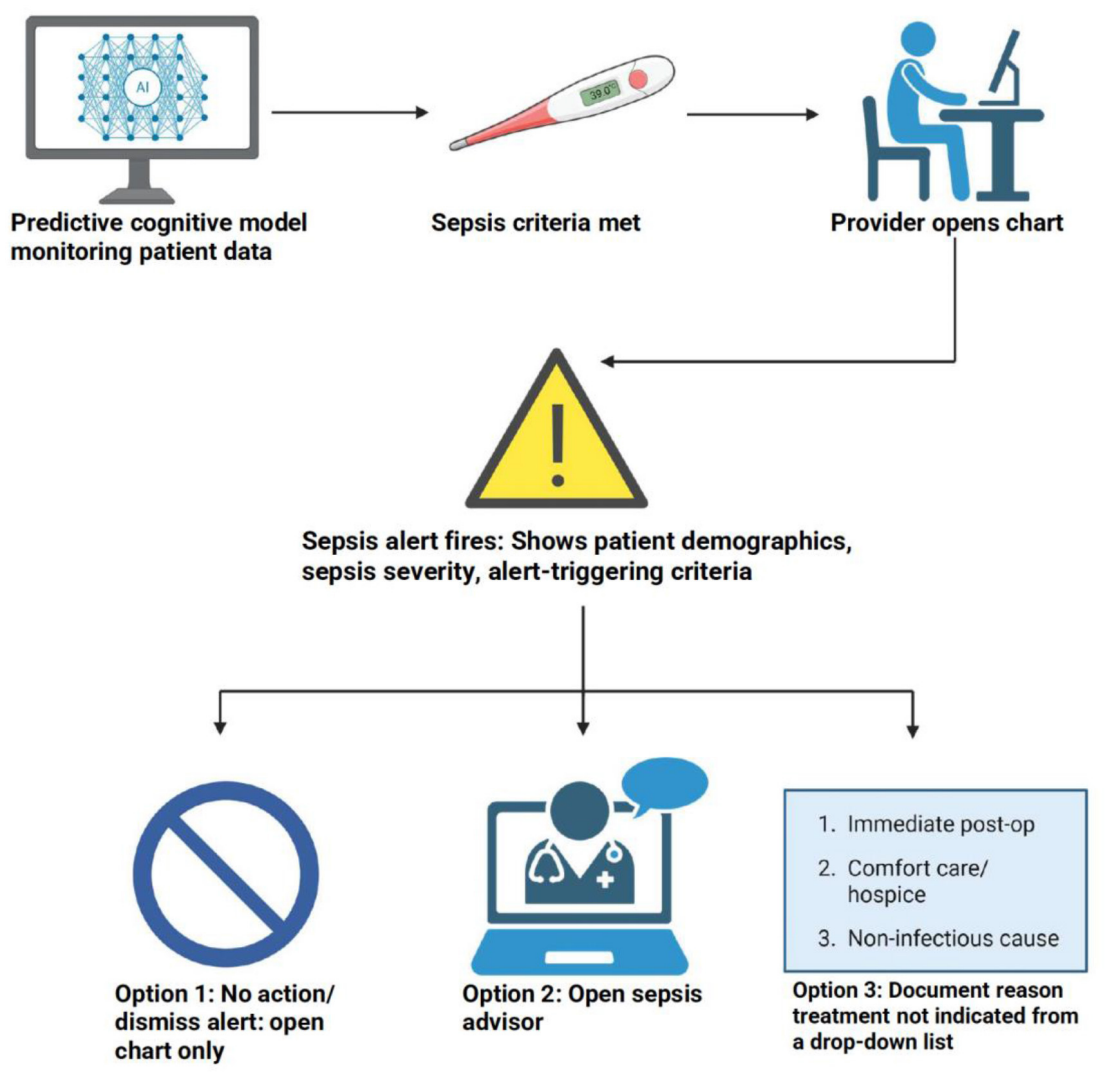
Flow diagram of the sepsis alert workflow showing data monitoring, alert firing, and three provider response options within the EHR. Created with BioRender.com.

The original version of this article has been updated.

