# Supplementary material for: Evaluation of sepsis CDS tool knowledge and utilization among graduate medical trainees: insights to inform redesign in an academic health system
Source: Front Med (Lausanne). 2026 Jan 5;12:1660390. doi: 10.3389/fmed.2025.1660390 (PMC12812634; doi:10.3389/fmed.2025.1660390)
Supplement: Supplementary file 1 [file Data_Sheet_1.docx]

# Supplementary Table S1. Full Survey Questions and Answer Options

1. Select your house staff position

- Resident

- Fellow

2. Select your PGY level

- PGY1

- PGY2

- PGY3

- PGY4

- PGY5

- PGY6

- PGY7

- PGY8

3. What is your gender?

- Male

- Female

- Non-binary

- Prefer not to say

4. What is your age?

- 20–29

- 30–39

- 40–49

- 50+

5. What department or division do you work in?

(Free text)

6. Are you familiar with the Inpatient Sepsis Alert?

- Yes

- No

- Not Sure

- I do not work inpatient

7. Have you ever used the embedded Sepsis Advisor CDS tool?

- I never used it as I was not aware that I could launch the advisor from the alert

- Yes

- No

8. How often do you utilize the sepsis advisor when alerted?

- Never

- Rarely

- Sometimes

- Always

9. Are you aware that the Sepsis Advisor CDS tool is available in the "add new order" section?

- Yes

- No

10. Which of the following best describes your experience? (select all that apply)

- I think it is alerting too much and contributes to alert fatigue

- I don't know how to use it or I was not aware that it had a CDS tool

- I didn't know that I can suppress the alert by selecting a drop-down option to exclude patient from sepsis

- I am frequently alerted by my patients' nurses regarding sepsis/SIRS alerts which is not clinically helpful

- I don't find it to be clinically useful for me or my patients

- I don't find it that bothersome and often do keep it in mind when reviewing a patient’s case

- Other (free text)

11. How helpful are the sepsis alert and the sepsis advisor?

- Both are unhelpful

- Sepsis alert is unhelpful but sepsis advisor is helpful

- Both are helpful / keep them as they are

- Sepsis alert is helpful but sepsis advisor is unhelpful

12. Since you find them unhelpful, which describes your experience? (select all that apply)

- The alert shouldn't pop when opening a patient's chart as this is the wrong workflow

- Often, my septic patients already have sepsis care orders in place making the alert redundant

- It often alerts in the wrong clinical context

- It alerts too frequently

- It is too cumbersome to use in a timely manner

- The advisor lacks orders or labs or medications important to patient care

- There's not a simple way to tell the alert that I am consulting and don't need the pop up

- Other (Free text)

13. Please indicate your suggestions (select all that apply)

- Add it as an asynchronous alert (not a pop-up)

- Remove the alert and change the advisor CDS tool into a PowerPlan only

- Add another option to the dropdown: "patient is already being adequately treated for sepsis."

- Change when the alert prompts (e.g., on placing or signing orders)

- Provide more education on how to use the alert/advisor

- Other (free text)

14. Recently, the sepsis alert was removed. How much do you agree?

- Strongly Agree

- Agree

- Neutral

- Disagree

- Strongly Disagree

15. Did you receive sepsis management education?

- Yes

- Yes, but have never been taught how to use the sepsis advisor

- No

- Not sure

16. I feel a well-designed and implemented sepsis CDS tool could enhance patient care

- Strongly Agree

- Agree

- Neutral

- Disagree

- Strongly Disagree

17. Do you know how to document sepsis care?

- Yes

- Yes, definitely

- I think so, but could use a refresher

- No

- I do not know what the sepsis bundle is

- No, definitely not

18. I document sepsis problems by: (select all that apply)

- Free texting my sepsis assessment and plan

- Adding sepsis to the "problem list"

- Using my own sepsis autotext

- Using the global sepsis autotext

- Using the current sepsis advisor

- Not applicable / I don't usually document about sepsis

- Other (free text)

19. OPTIONAL: If you would be willing to be contacted regarding your answers or opinions, please type your email AND/OR if you have additional suggestions on how to improve the sepsis alert, sepsis advisor.
